# Incidence, clinical characteristics and prognosis of tumor lysis syndrome following B-cell maturation antigen-targeted chimeric antigen receptor-T cell therapy in relapsed/refractory multiple myeloma

**DOI:** 10.3389/fimmu.2023.1125357

**Published:** 2023-05-04

**Authors:** Qiqi Zhang, Cheng Zu, Ruirui Jing, Youqin Feng, Yanlei Zhang, Mingming Zhang, Yuqi Lv, Jiazhen Cui, Linhui Zhou, Ye Meng, Linqin Wang, Zenan Cen, Alex H. Chang, Yongxian Hu, He Huang

**Affiliations:** ^1^ Bone Marrow Transplantation Center, the First Affiliated Hospital, Zhejiang University School of Medicine, Hangzhou, China; ^2^ Liangzhu Laboratory, Zhejiang University Medical Center, Hangzhou, China; ^3^ Institute of Hematology, Zhejiang University, Hangzhou, China; ^4^ Zhejiang Province Engineering Laboratory for Stem Cell and Immunity Therapy, Hangzhou, China; ^5^ Shanghai YaKe Biotechnology Ltd, Shanghai, China; ^6^ Clinical Translational Research Center, Shanghai Pulmonary Hospital, Tongji University School of Medicine, Shanghai, China

**Keywords:** B-cell maturation antigen, chimeric antigen receptor-T, multiple myeloma, tumor lysis syndrome, immunotherapy

## Abstract

**Background aims:**

B-cell maturation antigen (BCMA)-targeted chimeric antigen receptor-T cell (CAR-T) therapy is used for refractory or relapsed multiple myeloma (r/r MM). However, CAR-T-related tumor lysis syndrome (TLS) has been observed. We aimed to elucidate the incidence, clinical and laboratory characteristics, and prognosis of CAR-T cell-related TLS.

**Methods:**

Patients (n=105) with r/r MM treated with BCMA-targeted CAR-T cell therapy were included. Patient characteristics, laboratory parameters, and clinical outcomes were assessed.

**Results:**

Eighteen (17.1%) patients developed TLS after BCMA-targeted CAR-T cell therapy. The median time till TLS onset was 8 days. Patients with TLS had steep rise in uric acid (UA), creatinine, and lactate dehydrogenase (LDH) within 6 days following CAR-T cell infusion and presented earlier and persistent escalation of cytokines (C-reactive protein [CRP], interleukin-6 [IL-6], interferon-γ [IFN-γ], and ferritin levels). All 18 patients had cytokine release syndrome (CRS), of which 13 (72.2%) developed grade 3–4 CRS. Three of 18 patients (16.7%) developed immune effector cell-associated neurotoxicity syndrome (ICANS): two patients with grade 1 ICANS and one with grade 2 ICANS. TLS development had a negative effect on the objective response rate (77.8% in the TLS group vs. 95.4% in the non-TLS group, p<0.01). During the median follow-up of 15.1 months, the median PFS was poorer of patients with TLS (median: 3.4 months in the TLS group vs. 14.7 months in the non-TLS group, p<0.001, hazard ratio [HR]=3.5 [95% confidence interval [CI] 1.5–8.5]). Also, TLS development exhibited significant effects on OS (median: 5.0 months in the TLS group vs. 39.8 months in the non-TLS group, p<0.001, hazard ratio [HR]=3.7 [95% CI 1.3–10.3]). TLS was associated with a higher tumor burden, elevated baseline creatinine and UA levels, severe CRS, pronounced CAR-T cell expansion, and corticosteroid use.

**Conclusion:**

TLS is a frequently observed CAR-T therapy complication and negatively influences clinical response and prognosis. Close monitoring for TLS should be implemented during CAR-T cell therapy, especially for those at high TLS risk.

## Introduction

Chimeric antigen receptor-T cell (CAR-T) therapy has revolutionized the field of cancer treatment, especially in the paradigm of hematological malignancies. B-cell maturation antigen (BCMA)-targeted CAR-T therapy has shown remarkable efficacy and safety in patients with chemotherapy-refractory or relapsed multiple myeloma (r/r MM) in several clinical trials, with overall response rates of 50–98% and a complete response rate of 80% (1–3). However, treatment-related toxicities following CAR-T therapy remain a major obstacle to its broad clinical application. These include a life-threatening complication, tumor lysis syndrome (TLS), which has been reported with CD19-targeted CAR-T therapy. A previous study on CD19 CAR-T therapy has reported that 14.3% (2/14) of patients with chronic lymphoid leukemia (CLL) developed TLS during treatment. Another study of donor-derived allogeneic anti-CD19 CAR-T cells has also reported TLS in a progressive CLL patient with bulky adenopathy (4). However, data on CAR-T therapy-related TLS, which is a high-morbidity emergency requiring early recognition and prompt management, are limited. Hence, a comprehensive characterization of CAR-T therapy-associated TLS is urgently needed.

TLS results from the rapid destruction of tumor cells and massive release of intracellular components (such as potassium, phosphate, nucleic acids, cytokines) into the blood. TLS presents as a combination of metabolic disorders including hyperuricemia, hyperkalemia, hyperphosphatemia, and hypocalcemia, which lead to acute renal insufficiency, arrhythmia, epilepsy, and even sudden death (5, 6). MM is considered a low-proliferative malignancy with a rare incidence of TLS (7); however, with the emergence of novel therapeutic regimens in r/r MM, the incidence of TLS in MM has increased (8, 9).

Considering the distinct characteristics of CAR-T therapy-related TLS, we performed a *post-hoc* analysis of r/r MM to explore the incidence, clinical and laboratory characteristics as well as prognosis of CAR-T therapy-related TLS. This study aids early identification of patients at high risk of TLS during CAR-T cell therapy and the diagnosis and treatment of TLS.

## Methods

### Patients

This was a retrospective, single-institution analysis of patients with r/r MM treated with BCMA-targeted CAR-T cell therapy between July 2018 and December 2022 (n=99) (Chictr.org number, ChiCTR1800017404), and between June 2022 and August 2022 (n=6) (NCT05430945). The study was approved by the First Affiliated Hospital, School of Medicine, Zhejiang University Institutional Review Board and was conducted in accordance with the Declaration of Helsinki. All patients provided written informed consent to participate in the study. All patients received lymphodepletion chemotherapy with fludarabine 30 mg/m^2^ (days −4 to −2) and cyclophosphamide 500 mg/m^2^ (days −3 to −2), followed by CAR-T cell infusion on day 0. The manufacture of BCMA-targeted CAR-T cells has been described previously (10).

### Data collection

Patient, laboratory, and clinical data were obtained from the medical records of the First Affiliated Hospital, School of Medicine, Zhejiang University. All data collection and analyses were performed with approval from the Clinical Research Ethics Committee of the same institution.

### Toxicity evaluations

Cytokine release syndrome (CRS) was graded using the American Society for Transplantation and Cellular Therapy Consensus Grading for CRS (11). TLS was identified considering laboratory parameters and clinical manifestations according to the Howard criteria (5). Diagnosis of TLS requires at least two of the following metabolic abnormalities to be present during the same 24-hour period: uric acid (UA) level > 8.0 mg/dL (475.8 μmol/L) in adults or above the upper limit of the normal (ULN) range for age in children; phosphorus level > 4.5 mg/dL (1.5 mmol/L) in adults or > 6.5 mg/dL (2.1 mmol/L) in children; potassium level > 6.0 mmol/L; and corrected calcium level < 7.0 mg/dL (1.75 mmol/L) or ionized calcium level < 4.5 mg/dL (1.12 mmol/L). Clinical TLS according to the Howard criteria requires the presence of laboratory TLS in addition to any one of the following: increase of 0.3 mg/dL from baseline in serum creatinine level (or a single value > 1.5 times the ULN if no baseline creatinine level is available) or presence of oliguria defined as an average urine output < 0.5 mL/kg/h for 6 hours, cardiac dysrhythmia, seizures, or death either probably or definitely caused by the aforementioned metabolic abnormalities (5).

### Statistical analysis

Categorical parameters were compared using Fisher’s exact test or the χ^2^ test. Continuous parameters were compared using the Mann–Whitney U test. Kaplan–Meier curves for progression-free survival (PFS) and overall survival (OS) were compared using log-rank tests. Univariate analysis was performed using Fisher’s exact test or the χ^2^ test to describe associations between categorical variables. Statistical analyses and plots were generated using the Statistical Package for the Social Sciences (version 26) and R software (version 4.1.1).

## Results

### Incidence and characteristics of CAR-T therapy-related TLS

Of 105 patients who received BCMA-targeted CAR-T cell administration, 18 (17.1%) developed TLS (**Table 1**). The median time from CAR-T cell infusion to TLS onset was 8 days (range, 4–14).

**Table 1:** Clinical characteristics of patients with and without tumor lysis syndrome following B-cell maturation antigen-targeted chimeric antigen receptor-T cell therapy.

Characteristics	Total (n=105)	No TLS (n=87)	TLS (n=18)	*P* value
Sex, male, n (%)	62 (59.0%)	50 (57.5%)	12 (66.7%)	NS
Age (years), median (range)	60 (16–84)	60 (39–84)	59 (16–71)	NS
Types of myeloma, n (%)				
IgA	30 (28.6%)	26 (29.9%)	4 (22.2%)	NS
IgD	9 (8.6%)	7 (8.0%)	2 (11.1%)	
IgG	40 (38.1%)	36 (41.4%)	4 (22.2%)	
Light chain	25 (23.8%)	18 (20.7%)	7 (38.9%)	
Non-secretory	1 (1.0%)	1 (5.6%)	1 (5.9%)	
High-risk cytogenetic, n (%)	71 (67.6%)	58 (66.7%)	13 (72.2%)	NS
Durie–Salmon stage, n (%)				
I	3 (2.9%)	3 (3.4%)	0 (0%)	NS
II	11 (10.5%)	10 (11.5%)	1 (5.6%)	
III	84 (80.0%)	70 (80.5%)	14 (77.8%)	
Disease burden				
Percentage of plasma blasts in BM, median (IQR)	10.5(1.6, 38.0)	10.0 (1.2, 32.5)	31.5(3.1, 50.0)	<0.05
Extramedullary disease, (n%)	53 (50.5%)	45 (51.7%)	8 (44.4%)	NS
LDH (U/L), median (range)	194 (85–2240)	194 (194–2240)	199 (159–571)	NS
Prior therapy				
Previous lines of therapy, median (range)	3 (1–9)	3 (1–9)	3 (1–6)	NS
Previous ASCT	51 (48.6%)	39 (44.8%)	12 (66.7%)	NS
Baseline creatinine (μmol/L), median (IQR)	70.0 (57.0-90.0)	68.0 (55.5, 86.5)	92.5 (70.5, 160.5)	<0.01
Baseline uric acid (μmol/L), median (range)	319 (88–612)	313 (88–597)	428 (197–612)	<0.01
CRS				
Max grade 0	3 (2.9%)	3 (3.4%)	0 (0%)	<0.01
Max grade 1	17 (16.2%)	16 (18.4%)	1 (5.6%)	
Max grade 2	38 (36.2%)	34 (39.1%)	4 (22.2%)	
Max grade 3	43 (41.0%)	34 (39.1%)	9 (50.0%)	
Max grade 4	4 (3.8%)	0 (0%)	4 (22.2%)	
Toxicity management				
Received tocilizumab	37 (35.2%)	28 (32.2%)	9 (50.0%)	NS
Received corticosteroids	43 (41.0%)	12 (66.7%)	9 (50.0%)	<0.01

BM, bone marrow; IQR, interquartile range; CRS, cytokine release syndrome; ASCT, autologous stem cell transplant; LDH, lactate dehydrogenase. ns, not significant.

Patients with TLS had hyperuricemia, hyperkalemia, hyperphosphatemia, hypocalcemia, and elevated lactate dehydrogenase (LDH) levels (12). All 18 patients developed renal dysfunction, indicated by elevated serum creatinine levels, oliguria, or anuria. Eight patients (47.1%) had arrhythmia, mostly presenting as atrial fibrillation. All 18 patients had CRS: 13 (72.2%) developed grade 3–4 CRS. The median time to CRS onset of any grade was 1 day (range, 1–17). Two patients developed TLS before CRS; remaining developed TLS after CRS. Three of 18 patients (16.7%) developed immune effector cell-associated neurotoxicity syndrome (ICANS): two patients with grade 1 ICANS and one with grade 2 ICANS.

To identify clinical and laboratory features associated with TLS in r/r MM patients after BCMA-targeted CAR-T cell therapy, we examined demographic characteristics, baseline and post-infusion clinical features, and laboratory findings (**Table 1**). Baseline was defined as the time point before administration of lymphodepleting drugs. Univariate analysis revealed that elevated baseline creatinine and UA levels, and disease burden correlated with TLS development (12). We did not identify differences in demographic features, disease subtypes, cytogenetic status, Durie–Salmon stage, and prior therapy between those with and without TLS. Of note, the maximum CRS grade after CAR-T cell infusion was significantly different between patients with and without TLS.

The anti-interleukin (IL)-6 receptor monoclonal antibody tocilizumab and/or corticosteroids have been used to mitigate TLS. Seven (38.9%) of 18 patients received tocilizumab plus corticosteroids; two (11.1%), tocilizumab alone; and five (27.8%), corticosteroid alone. Corticosteroid use was positively associated with TLS, whereas tocilizumab use indicated no difference. Of 18 patients with renal dysfunction, 5 received continuous renal replacement therapy for the median duration of 6.5 days (range, 3–23).

### Clinical outcomes

**Figure 1:**
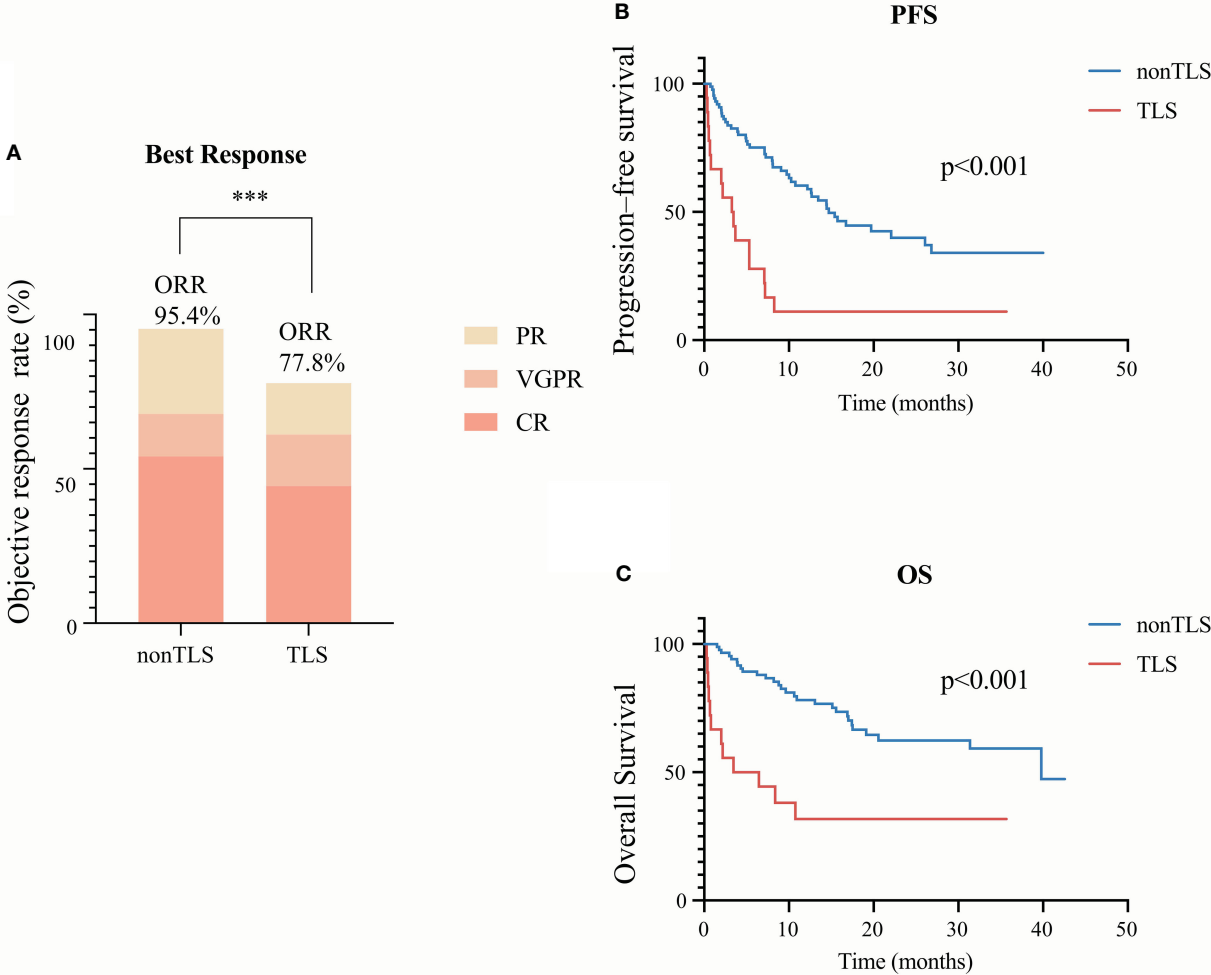
Clinical outcomes of tumor lysis syndrome (TLS) in patients receiving B-cell maturation antigen-targeted chimeric antigen receptor-T cell (CAR-T) therapy. **(A)** Best clinical response 1 month after CAR-T infusion. **(B)** Kaplan–Meier estimate of progression-free survival of patients with or without TLS. **(C)** Kaplan–Meier estimate of overall survival of patients with and without TLS. *** means “p value<0.001”.

Overall, 55 (52.4%) patients achieved complete remission (CR) with BCMA-targeted CAR-T therapy. Fifteen (14.3%) patients had very-good-partial remission (VGPR), and 27 (25.7%) had partial remission (PR). Among 18 patients with TLS, 8 (47.0%) achieved CR, 3 (17.6%) had VGPR, 3 (11.8%) had PR, and the remaining 4 (23.5%) had progressive disease. TLS development had a negative effect on the objective response rate (p<0.01) (**Figure 1A**).

During the median follow-up of 15.1 months, the median PFS was poorer of patients with TLS (median: 3.4 months in the TLS group vs. 14.7 months in the non-TLS group, p<0.001, hazard ratio [HR]=3.5 [95% confidence interval [CI] 1.5–8.5]) (**Figure 1B**). Also, TLS development exhibited significant effects on OS (median: 5.0 months in the TLS group vs. 39.8 months in the non-TLS group, p<0.001, hazard ratio [HR]=3.7 [95% CI 1.3–10.3]) (**Figure 1C**).

### Temporal changes of laboratory findings in patients with TLS

**Figure 2:**
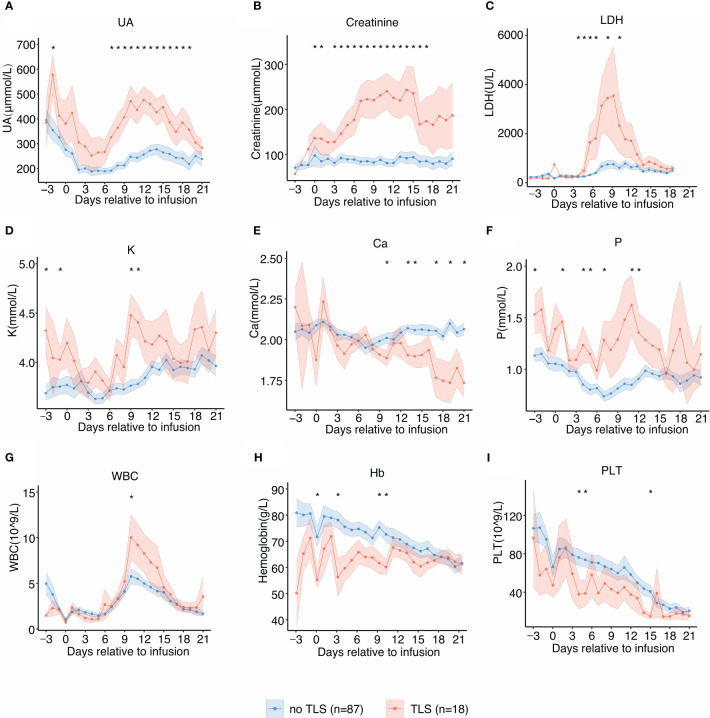
Laboratory values of tumor lysis syndrome (TLS)-related biomarkers and trends relative to B-cell maturation antigen-targeted chimeric antigen receptor-T cell (CAR-T) infusion. Each dot indicates the mean value at a different time point. The error bands indicate the estimated 68% confidence interval. Asterisks denote p<0.05 per the Mann–Whitney U test. **(A)** Uric acid (days −3 to 21). **(B)** Creatinine (days −3 to 21). **(C)** Lactate dehydrogenase (LDH) (days −3 to 21). **(D)** Potassium (K) (days −3 to 21). **(E)** Calcium (Ca) (days −3 to 21). **(F)** Phosphate (P) (days −3 to 21). **(G)** White blood cell counts (days −3 to 21). **(H)** Hemoglobin (days −3 to 21). **(I)** Platelet counts (days −3 to 21).

The temporal dynamics of the laboratory parameters associated with TLS during the clinical course was in agreement with previous reports. Patients with TLS had prolonged and pronounced hyperuricemia, hyperkalemia, hyperphosphatemia, hypocalcemia, and elevated levels of serum LDH after CAR-T infusion (**Figures 2A–F**) when compared with those without TLS. UA, creatinine, potassium, and phosphate levels were persistently elevated from day 6 post-infusion to approximately 3 weeks post-infusion, while calcium levels remained low from day 9 post-infusion to 3 weeks post-infusion. LDH exhibited an intermittent increase from days 4 to 14 in patients with TLS. Patients with TLS had higher white blood cell count, lower platelet count, and lower hemoglobin levels than those without TLS (**Figures 2G–I**).

### Dynamics of cytokines during treatment

**Figure 3:**
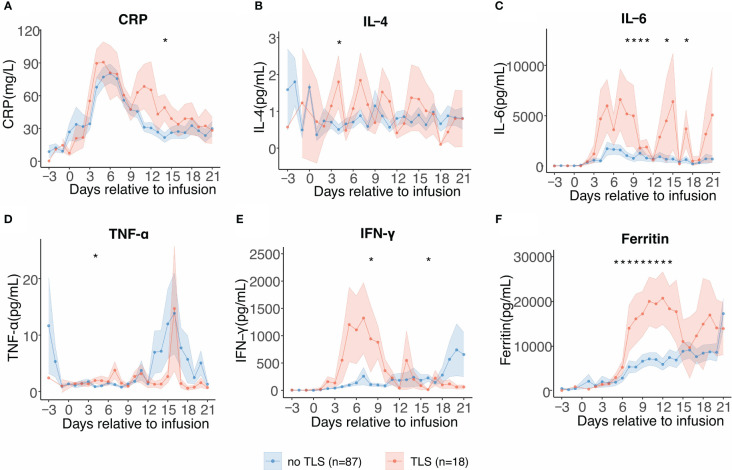
Serial cytokine profiling relative to B-cell maturation antigen-targeted chimeric antigen receptor-T cell (CAR-T) infusion. Each dot indicates the mean value of each time point. The error bands indicate the estimated 68% confidence interval. Asterisks denote p<0.05 per the Mann–Whitney U test. **(A)** C-reactive protein (days −3 to 21). **(B)** Interleukin-4 (IL-4) (days −3 to 21). **(C)** Interleukin-6 (IL-6) (days −3 to 21). **(D)** Tumor necrosis factor-α (TNF-α) (days −3 to 21). **(E)** Interferon-γ (INF-γ) (days −3 to 21). **(F)** Ferritin (days −3 to 21).

As we observed a significant difference in CRS grades in patients with and without TLS, we performed serial cytokine profiling to study the inflammatory patterns between the two groups. Patients who developed TLS had earlier and more persistent escalation of cytokine levels, suggesting early and profound systemic inflammation. In patients with TLS, several inflammatory cytokines including C-reactive protein (CRP), IL-6, interferon-γ (IFN-γ), and ferritin levels were persistently elevated from days 5 to 18, and these cytokines showed biphasic lifting-up when compared with those in patients without TLS (**Figures 3A–F**). Contrastingly, tumor necrosis factor-α level increased in the later stages and exhibited no significant differences in patients with TLS. No significant difference in the expression pattern of IL-4 was observed. Although the etiology of TLS following CAR-T therapy is unclear, cytokines and TLS may exhibit a complex interplay.

### CAR-T cell expansion in TLS

**Figure 4:**
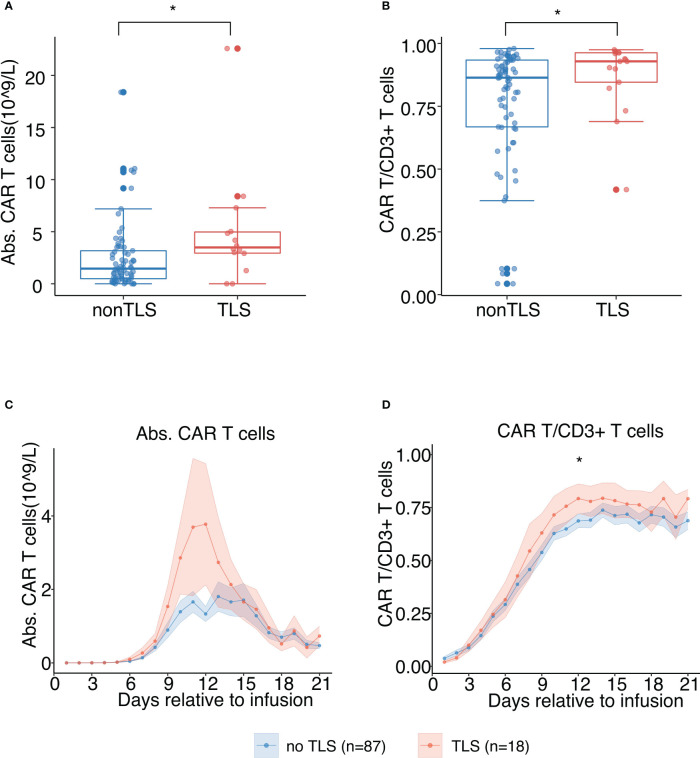
*In vivo* chimeric antigen receptor-T cell (CAR-T) cell expansion following B-cell maturation antigen-targeted CAR-T therapy for patients with and without TLS. **(A)** Peak values of absolute counts of peripheral CAR-T cells. The scatter plot depicts all values, and lines represent median and interquartile range (IQR). **(B)** Peak values of proportions of peripheral CAR-T cells in CD^3+^ T cells. The scatter plot depicts all values, and lines represent median and IQR. **(C)** Absolute counts of peripheral CAR-T cells post CAR-T infusion (days 1 to 21). Each dot indicates the mean value of each time point. The error bands indicate the estimated 68% confidence interval. **(D)** Proportion of peripheral CAR-T cells in CD^3+^ T cells post CAR-T infusion (days 1 to 21). Each dot indicates the mean value at a different time point. The error bands indicate the estimated 68% confidence interval. All asterisks denote p<0.05 per the Mann–Whitney U test.

Higher CAR-T cell expansion (absolute CAR-T cell count and its proportion in CD3^+^ T cells) was observed in patients with TLS, indicating that prominent CAR-T cell expansion positively correlates with a higher risk of TLS following CAR-T therapy (p<0.01) (**Figures 4A, B**). The absolute peripheral CAR-T cell count and its proportion in peripheral CD3^+^ T cells were predominantly higher in patients with TLS. The time point at which we noted a rise in CAR-T cell count coincided with TLS onset time, suggesting causality between CAR-T cell expansion and tumor lysis (**Figures 4C, D**). This did however not reach statistical significance, considering the limited sample size.

## Discussion

CAR-T therapy has been promising, especially in r/r hematological malignancies (2, 13, 14). Recognizing CAR-T cell-related toxicities is substantial for improving its clinical application (15). Several TLS cases after CD19 CAR-T therapy have been observed (4, 16); however, TLS in the context of BCMA-targeted CAR-T therapy has not been reported, and recognizing TLS as a vital CAR-T cell-related toxicity is imperative. Here, we present the most comprehensive clinical and laboratory characterization of TLS following BCMA-targeted CAR-T cell therapy.

In our cohort, TLS incidence following BCMA-targeted CAR-T therapy was 17.1%, indicating that TLS is a relatively common complication in r/r MM treated with CAR-T cells, similar to the previously reported incidence of CD19 CAR-T therapy (4, 16). However, TLS incidence in MM is higher than that with other therapeutics (8, 9). Additionally, in accordance with previous studies, TLS following CAR-T therapy showed detrimental effects on patient prognosis, clinical response, PFS and OS. Acute kidney injury (AKI) had high prevalence during the acute phase of TLS and was reported as a major cause of death in TLS with an OR of 10.41 for in-hospital mortality (17–19). In our cohort, TLS related AKI resulted in ICU administration, renal replacement therapy and 30-day modality. Arrhythmia secondary to TLS was also detrimental. Cardiac arrest and sudden death were reported due to TLS (20).

CAR-T cells exhibit a unique profile of toxicity as cellular products. We explored temporal changes in TLS-related laboratory examinations and observed that patients with TLS presented with persistent hyperuricemia, hyperkalemia, hyperphosphatemia, and hypocalcemia after CAR-T infusion. Occasional late-onset TLS was also noted. Considering we noticed that the TLS onset coincides with the rise in the CAR-T cell count-time curve, these findings could be interpreted as delayed CAR-T cell expansion and ongoing anti-myeloma efficacy.

Previous studies have identified several TLS risk factors in MM such as older age, high disease burden, myeloma cells in peripheral blood, bortezomib use, impaired renal function, and elevated baseline UA (9, 21); however, the established predictive markers may have overlooked the potential pathophysiological influence of CAR-T cells. As cellular products, CAR-T cells exhibit a unique profile of toxicities distinct from those of chemotherapy drugs. In our cohort, higher tumor burden, elevated creatinine and UA levels, severe CRS grade, prominent CAR-T expansion, and corticosteroid use were positively associated with TLS on univariate analysis. High disease burden, renal dysfunction, kidney involvement and hyperuricemia are established risk factors for TLS (7, 9, 21), whereas CRS and CAR-T expansion are newly uncovered risk factors for CAR-T-related TLS, the underlying relationship of which is worth further investigation.

Our findings suggest that patients with severe CRS are predisposed to developing TLS. It is reasonable to hypothesize that patients with high tumor burden tend to develop CRS and TLS simultaneously, as disease burden is well recognized contributing factor for both CRS (22, 23) and TLS (7, 24). Besides, our observations suggested that CAR-T cell expansion is associated with TLS. Higher expansion of CAR-T cells is also associated with both CRS (25), thus we suppose patients with predominant CAR-T cell expansion are likely to develop CRS and TLS simultaneously. We postulate that although profound CAR-T cell expansion produces superior anti-tumor efficacy, it may magnify the risk of toxicities, including CRS and TLS.

But beyond all that, CRS and TLS may have a complex interplay that needs to be elucidated. CRS etiology has not yet been fully elucidated. Current hypotheses include inflammatory cytokines and chemokines released by CAR-T cells, activated monocytes and macrophages, endothelial injury, vascular leakage and subsequent acute systemic inflammatory symptoms, and secondary organ dysfunction (26, 27). On the one hand, the intracellular substances released during tumor cell lysis may promote a systematic inflammatory response and trigger CRS. On the other hand, strikingly elevated cytokines in CRS may contribute to TLS development, as we noted a dramatic increase in IL-6, INF-γ, and ferritin levels during TLS, and these cytokines trigger inflammatory cell death, apoptosis, and pyroptosis (27–29). Elevation of serum IL-6 levels was reported in a patient with Hodgkin’s lymphoma who developed TLS after chemotherapy (30). Recently, Supiot et al. reported that a patient with MM simultaneously developed TLS and CRS after radiotherapy (31). Systemic inflammatory response syndrome and multiple organ dysfunction were observed in patients with TLS and hematological malignancies or solid tumors (32).

Of note, an association between corticosteroid use and TLS was observed in our study, although the causal relationship could not be determined since not all patients received corticosteroids before TLS onset. Corticosteroids are widely administered to manage CAR-T cell-related toxicities, and studies have shown that prophylactic use and earlier dosing have the potential to reduce the incidence of severe CRS and neurologic events (33, 34). However, concerns have been raised postulating the possible role of steroids in increasing TLS risk. Steroids have anti-tumor cytotoxicity and are widely used as components of chemotherapy in the frontline treatment of hematologic malignancies (35). Corticosteroid-induced TLS has been reported in patients with hematological malignancies and solid tumors (36–40). Although TLS rarely occurs after single-agent corticosteroid administration, clinicians prescribing corticosteroids to patients should be aware of this life-threatening complication.

The potential morbidity resulting from TLS necessitates prophylactic measures for at-risk patients. Recognizing risk factors and closely monitoring electrolytes, renal function, and cytokine levels can prevent TLS (24). Major prevention strategies include hydration and UA level control (24). For patients with elevated plasma UA levels, current recommendations for TLS prophylaxis include allopurinol (24), rasburicase (41), and febuxostat (42). Our novel findings suggest that controlling CRS reduces TLS risk. For those with severe CRS or cytokine hypersecretion, controlling CRS or administering cytokine inhibitors may help prevent subsequent TLS. Ruxolitinib, a Janus kinase (JAK)1/JAK2 inhibitor, can downregulate pro-inflammatory cytokine signaling (43, 44). Another JAK1 inhibitor, itacitinib, also reduced cytokines such as IFN-γ and IL-6 in CAR-T-induced CRS in a preclinical study (45). Additionally, the IL-6 receptor antagonist tocilizumab has been widely used to treat CAR-T cell-related toxicities, especially CRS (46, 47).

In the setting of CAR-T related TLS in MM, most patients manifested with renal dysfunction. Aggressive hydration, management of hyperuricemia, and hemodialysis for severe renal failure are recommended (24). For patients with electrolytic disturbances, treating hyperphosphatemia, hyperkalemia, and hypocalcemia is necessary. Additionally, antiarrhythmics are required for confirmed arrhythmias. Prompt intervention to correct hyperkalemia and hypocalcemia is also paramount to manage cardiac arrhythmias. Alkalinization is currently not recommended because unequivocal evidence of efficacy is lacking (7).

In conclusion, implementing prophylaxis for TLS during CAR-T therapy is reasonable, especially for patients with high disease burden and pre-existing renal insufficiency and for those experiencing severe CRS during the course. Clinicians should closely monitor electrolytes, renal function and cytokines during CAR-T therapy to prevent life-threatening TLS.

## Data availability statement

The original contributions presented in the study are included in the article/supplementary material. Further inquiries can be directed to the corresponding authors.

## Ethics statement

The studies involving human participants were reviewed and approved by Clinical Research Ethics Committee of the First Affiliated Hospital, School of Medicine, Zhejiang University. Written informed consent to participate in this study was provided by the participants’ legal guardian/next of kin.

## Author contributions

QZ, YH and HH designed the study. QZ, CZ, RJ, YF, YM and JC analyzed and interpreted the data. QZ, CZ, RJ, YH, and HH drafted the article. LZ, YM, LW, and CZ revised the article. AC and YZ helped the manufacture of CAR-T cells. YH, MZ, CZ and HH provided CAR-T cell treatment and care to patients. All authors contributed to the article and approved the submitted version.

## References

[1] Raje N Berdeja J Lin Y Siegel D Jagannath S Madduri D. Anti-BCMA CAR T-cell therapy bb2121 in relapsed or refractory multiple myeloma [J]. N Engl J Med (2019) 380(18):1726–37. doi: 10.1056/NEJMoa1817226 PMC8202968 31042825

[2] Berdeja JG Madduri D Usmani SZ Jakubowiak A Agha M Cohen AD. Ciltacabtagene autoleucel, a b-cell maturation antigen-directed chimeric antigen receptor T-cell therapy in patients with relapsed or refractory multiple myeloma (CARTITUDE-1): a phase 1b/2 open-label study [J]. Lancet (2021) 398(10297):314–24. doi: 10.1016/S0140-6736(21)00933-8 34175021

[3] Munshi NC Anderson LD Jr. Shah N Madduri D Berdeja J Lonial S. Idecabtagene vicleucel in relapsed and refractory multiple myeloma [J]. N Engl J Med (2021) 384(8):705–16. doi: 10.1056/NEJMoa2024850 33626253

[4] Kochenderfer JN Dudley ME Carpenter RO Kassim SH Rose JJ Telford WG. Donor-derived CD19-targeted T cells cause regression of malignancy persisting after allogeneic hematopoietic stem cell transplantation [J]. Blood (2013) 122(25):4129–39. doi: 10.1182/blood-2013-08-519413 PMC3862276 24055823

[5] Howard SC Jones DP Pui CH. The tumor lysis syndrome [J]. N Engl J Med (2011) 364(19):1844–54. doi: 10.1056/NEJMra0904569 PMC3437249 21561350

[6] Gupta A Moore JA. Tumor lysis syndrome [J]. JAMA Oncol (2018) 4(6):895. doi: 10.1001/jamaoncol.2018.0613 29801143

[7] Cairo MS Coiffier B Reiter A Younes A Panel TLSE. Recommendations for the evaluation of risk and prophylaxis of tumour lysis syndrome (TLS) in adults and children with malignant diseases: an expert TLS panel consensus [J]. Br J Haematol (2010) 149(4):578–86. doi: 10.1111/j.1365-2141.2010.08143.x 20331465

[8] Singh A Gupta S Yim B Thekkekara R. Tumor lysis syndrome in multiple myeloma: an increasingly recognized risk-a report of seven cases [J]. Indian J Hematol Blood Transfus (2017) 33(1):41–4. doi: 10.1007/s12288-016-0731-6 PMC5280866 28194054

[9] Kondo M Hotta Y Yamauchi K Sanagawa A Komatsu H Iida S. Bortezomib administration is a risk factor associated with the development of tumor lysis syndrome in male patients with multiple myeloma: a retrospective study [J]. BMC Cancer (2020) 20(1):1117. doi: 10.1186/s12885-020-07592-9 33203424 PMC7672870

[10] Zhang M Zhou L Zhao H Zhang Y Wei G Hong R. Risk factors associated with durable progression-free survival in patients with relapsed or refractory multiple myeloma treated with anti-BCMA CAR T-cell therapy [J]. Clin Cancer Res (2021) 27(23):6384–92. doi: 10.1158/1078-0432.CCR-21-2031 PMC9401500 34548316

[11] Lee DW Santomasso BD Locke FL Ghobadi A Turtle CJ Brudno JN. ASTCT consensus grading for cytokine release syndrome and neurologic toxicity associated with immune effector cells [J]. Biol Blood Marrow Transplant (2019) 25(4):625–38. doi: 10.1016/j.bbmt.2018.12.758 PMC12180426 30592986

[12] Zhang Q Zu C Meng Y Lyu Y Hu Y Huang H. Risk factors of tumor lysis syndrome in relapsed/refractory multiple myeloma patients undergoing BCMA CAR-T cell therapy [J]. Zhejiang Da Xue Xue Bao Yi Xue Ban (2022) 51(2):144–50. doi: 10.3724/zdxbyxb-2022-0038 PMC9353642 36161293

[13] Schuster SJ Tam CS Borchmann P Worel N McGuirk JP Holte H. Long-term clinical outcomes of tisagenlecleucel in patients with relapsed or refractory aggressive b-cell lymphomas (JULIET): a multicentre, open-label, single-arm, phase 2 study [J]. Lancet Oncol (2021) 22(10):1403–15. doi: 10.1016/S1470-2045(21)00375-2 34516954

[14] Locke FL Miklos DB Jacobson CA Perales MA Kersten MJ Oluwole OO. Axicabtagene ciloleucel as second-line therapy for Large b-cell lymphoma [J]. N Engl J Med (2022) 386(7):640–54. doi: 10.1056/NEJMoa2116133 34891224

[15] Schubert ML Schmitt M Wang L Ramos CA Jordan K Muller-Tidow C. Side-effect management of chimeric antigen receptor (CAR) T-cell therapy [J]. Ann Oncol (2021) 32(1):34–48. doi: 10.1016/j.annonc.2020.10.478 33098993

[16] Porter DL Hwang WT Frey NV Lacey SF Shaw PA Loren AW. Chimeric antigen receptor T cells persist and induce sustained remissions in relapsed refractory chronic lymphocytic leukemia [J]. Sci Transl Med (2015) , 7(303):303ra139. doi: 10.1126/scitranslmed.aac5415 PMC5909068 26333935

[17] Barbar T Jaffer SI. Tumor lysis syndrome [J]. Adv Chronic Kidney Dis (2021) 28(5):438–46 e1. doi: 10.1053/j.ackd.2021.09.007 35190110

[18] Darmon M Guichard I Vincent F Schlemmer B Azoulay E. Prognostic significance of acute renal injury in acute tumor lysis syndrome [J]. Leuk Lymphoma (2010) 51(2):221–7. doi: 10.3109/10428190903456959 20001238

[19] Abdel-Nabey M Chaba A Serre J Lengline E Azoulay E Darmon M. Tumor lysis syndrome, acute kidney injury and disease-free survival in critically ill patients requiring urgent chemotherapy [J]. Ann Intensive Care (2022) 12(1):15. doi: 10.1186/s13613-022-00990-1 35166948 PMC8847484

[20] Roberts AW Davids MS Pagel JM Kahl BS Puvvada SD Gerecitano JF. Targeting BCL2 with venetoclax in relapsed chronic lymphocytic leukemia [J]. N Engl J Med (2016) 374(4):311–22. doi: 10.1056/NEJMoa1513257 PMC7107002 26639348

[21] Oiwa K Morita M Kishi S Okura M Tasaki T Matsuda Y. High risk of tumor lysis syndrome in symptomatic patients with multiple myeloma with renal dysfunction treated with bortezomib [J]. Anticancer Res (2016) 36(12):6655–62. doi: 10.21873/anticanres.11274 27919998

[22] Hay KA Hanafi LA Li D Gust J Liles WC Wurfel MM. Kinetics and biomarkers of severe cytokine release syndrome after CD19 chimeric antigen receptor-modified T-cell therapy [J]. Blood (2017) 130(21):2295–306. doi: 10.1182/blood-2017-06-793141 PMC5701525 28924019

[23] Wang J Hu Y Yang S Wei G Zhao X Wu W. Role of fluorodeoxyglucose positron emission Tomography/Computed tomography in predicting the adverse effects of chimeric antigen receptor T cell therapy in patients with non-Hodgkin lymphoma [J]. Biol Blood Marrow Transplant (2019) 25(6):1092–8. doi: 10.1016/j.bbmt.2019.02.008 30769193

[24] Coiffier B Altman A Pui CH Younes A Cairo MS. Guidelines for the management of pediatric and adult tumor lysis syndrome: an evidence-based review [J]. J Clin Oncol (2008) 26(16):2767–78. doi: 10.1200/JCO.2007.15.0177 18509186

[25] Mueller KT Maude SL Porter DL Frey N Wood P Han X. Cellular kinetics of CTL019 in relapsed/refractory b-cell acute lymphoblastic leukemia and chronic lymphocytic leukemia [J]. Blood (2017) 130(21):2317–25. doi: 10.1182/blood-2017-06-786129 PMC5731220 28935694

[26] Morris EC Neelapu SS Giavridis T Sadelain M. Cytokine release syndrome and associated neurotoxicity in cancer immunotherapy [J]. Nat Rev Immunol (2022) 22(2):85–96. doi: 10.1038/s41577-021-00547-6 34002066 PMC8127450

[27] Li X Shao M Zeng X Qian P Huang H. Signaling pathways in the regulation of cytokine release syndrome in human diseases and intervention therapy [J]. Signal Transduct Target Ther (2021) 6(1):367. doi: 10.1038/s41392-021-00764-4 34667157 PMC8526712

[28] Karki R Sharma BR Tuladhar S Williams EP Zalduondo L Samir P. Synergism of TNF-alpha and IFN-gamma triggers inflammatory cell death, tissue damage, and mortality in SARS-CoV-2 infection and cytokine shock syndromes [J]. Cell (2021) 184(1):149–68 e17. doi: 10.1016/j.cell.2020.11.025 33278357 PMC7674074

[29] Liu Y Fang Y Chen X Wang Z Liang X Zhang T. Gasdermin e-mediated target cell pyroptosis by CAR T cells triggers cytokine release syndrome [J]. Sci Immunol (2020) 5(43):eaax7969. doi: 10.1126/sciimmunol.aax7969 31953257

[30] Suzuki T Takeuchi M Saeki H Yamazaki S Koga H Abe D. Super-acute onset of tumor lysis syndrome accompanied by hypercytokinemia during treatment of hodgkin's lymphoma with ABVD chemotherapy [J]. Clin Ther (2010) 32(3):527–31. doi: 10.1016/j.clinthera.2010.03.010 20399989

[31] Cailleteau A Touzeau C Jamet B Guimas V Jouglar E Supiot S. Cytokine release syndrome and tumor lysis syndrome in a multiple myeloma patient treated with palliative radiotherapy: a case report and review of the literature [J]. Clin Transl Radiat Oncol (2022) 32:24–8. doi: 10.1016/j.ctro.2021.11.004 PMC8591462 34816023

[32] Soares M Feres GA Salluh JI. Systemic inflammatory response syndrome and multiple organ dysfunction in patients with acute tumor lysis syndrome [J]. Clinics (Sao Paulo) (2009) 64(5):479–81. doi: 10.1590/S1807-59322009000500016 PMC2694253 19488615

[33] Oluwole OO Bouabdallah K Munoz J De Guibert S Vose JM Bartlett NL. Prophylactic corticosteroid use in patients receiving axicabtagene ciloleucel for large b-cell lymphoma [J]. Br J Haematol (2021) 194(4):690–700. doi: 10.1111/bjh.17527 34296427 PMC8457222

[34] Topp MS Van Meerten T Houot R Minnema MC Bouabdallah K Lugtenburg PJ. Earlier corticosteroid use for adverse event management in patients receiving axicabtagene ciloleucel for large b-cell lymphoma [J]. Br J Haematol (2021) 195(3):388–98. doi: 10.1111/bjh.17673 PMC9293158 34590303

[35] Burwick N Sharma S. Glucocorticoids in multiple myeloma: past, present, and future [J]. Ann Hematol (2019) 98(1):19–28. doi: 10.1007/s00277-018-3465-8 30073393

[36] Duzova A Cetin M Gumruk F Yetgin S. Acute tumour lysis syndrome following a single-dose corticosteroid in children with acute lymphoblastic leukaemia [J]. Eur J Haematol (2001) 66(6):404–7. doi: 10.1034/j.1600-0609.2001.066006404.x 11488940

[37] Vaisban E Zaina A Braester A Manaster J Horn Y. Acute tumor lysis syndrome induced by high-dose corticosteroids in a patient with chronic lymphatic leukemia [J]. Ann Hematol (2001) 80(5):314–5. doi: 10.1007/s002770000276 11446738

[38] Sparano J Ramirez M Wiernik PH. Increasing recognition of corticosteroid-induced tumor lysis syndrome in non-hodgkin's lymphoma [J]. Cancer (1990) 65(5):1072–3. doi: 10.1002/1097-0142(19900301)65:5<1072::AID-CNCR2820650504>3.0.CO;2-A 2302658

[39] Van De Kerkhof JJ Peters WG Visser J Creemers GJ. Acute tumor lysis syndrome in a patient with multiple myeloma treated with dexamethasone monotherapy [J]. Neth J Med (2001) 59(2):83–5. doi: 10.1016/S0300-2977(01)00132-2 11550657

[40] Kim JO Jun DW Tae HJ Lee KN Lee HL Lee OY. Low-dose steroid-induced tumor lysis syndrome in a hepatocellular carcinoma patient [J]. Clin Mol Hepatol (2015) 21(1):85–8. doi: 10.3350/cmh.2015.21.1.85 PMC4379202 25834806

[41] Cortes J Moore JO Maziarz RT Wetzler M Craig M Matous J. Control of plasma uric acid in adults at risk for tumor lysis syndrome: efficacy and safety of rasburicase alone and rasburicase followed by allopurinol compared with allopurinol alone–results of a multicenter phase III study [J]. J Clin Oncol (2010) 28(27):4207–13. doi: 10.1200/JCO.2009.26.8896 PMC4979236 20713865

[42] Spina M Nagy Z Ribera JM Federico M Aurer I Jordan K. FLORENCE: a randomized, double-blind, phase III pivotal study of febuxostat versus allopurinol for the prevention of tumor lysis syndrome (TLS) in patients with hematologic malignancies at intermediate to high TLS risk [J]. Ann Oncol (2015) 26(10):2155–61. doi: 10.1093/annonc/mdv317 26216382

[43] Yeleswaram S Smith P Burn T Covington M Juvekar A Li Y. Inhibition of cytokine signaling by ruxolitinib and implications for COVID-19 treatment [J]. Clin Immunol (2020) 218:108517. doi: 10.1016/j.clim.2020.108517 32585295 PMC7308779

[44] Keenan C Nichols KE Albeituni S. Use of the JAK inhibitor ruxolitinib in the treatment of hemophagocytic lymphohistiocytosis [J]. Front Immunol (2021) 12:614704. doi: 10.3389/fimmu.2021.614704 33664745 PMC7923355

[45] Huarte E O'connor RS Peel MT Nunez-Cruz S Leferovich J Juvekar A. Itacitinib (INCB039110), a JAK1 inhibitor, reduces cytokines associated with cytokine release syndrome induced by CAR T-cell therapy [J]. Clin Cancer Res (2020) 26(23):6299–309. doi: 10.1158/1078-0432.CCR-20-1739 PMC7895329 32998963

[46] Ali SA Shi V Maric I Wang M Stroncek DF Rose JJ. T Cells expressing an anti-b-cell maturation antigen chimeric antigen receptor cause remissions of multiple myeloma [J]. Blood (2016) 128(13):1688–700. doi: 10.1182/blood-2016-04-711903 PMC5043125 27412889

[47] Schuster SJ Svoboda J Chong EA Nasta SD Mato AR Anak O. Chimeric antigen receptor T cells in refractory b-cell lymphomas [J]. N Engl J Med (2017) 377(26):2545–54. doi: 10.1056/NEJMoa1708566 PMC5788566 29226764

